# Genome-wide identification and characterization of GATA family genes in wheat

**DOI:** 10.1186/s12870-022-03733-3

**Published:** 2022-07-27

**Authors:** Xue Feng, Qian Yu, Jianbin Zeng, Xiaoyan He, Wenxing Liu

**Affiliations:** 1 College of Agronomy, Qingdao, Agricultural University, Qingdao, 266109 China; 2 The Key Laboratory of the Plant Development and Environmental Adaptation Biology, inistry of Education, School of Life Sciences, Shandong University, Shandong Province, Qingdao, 266237 China

**Keywords:** Wheat (*Triticum aestivum* L.), GATA,Genome-wide, Abiotic stress, Expression patterns

## Abstract

**Background:**

Transcription factors GATAs were a member of zinc finger protein, which could bind DNA regulatory regions to control expression of target genes, thus influencing plant growth and development either in normal condition or environmental stresses. Recently, *GATA* genes have been found and functionally characterized in a number of plant species. However, little information of *GATA* genes were annotated in wheat.

**Results:**

In the current study, 79 *GATA* genes were identified in wheat, which were unevenly located on 21 chromosomes. According to the analysis of phylogenetic tree and functional domain structures, *TaGATAs* were classified into four subfamilies (I, II, III, and IV), consist of 35, 21, 12, and 11 genes, respectively. Meanwhile, the amino acids of 79 TaGATAs exhibited apparent difference in four subfamilies according to GATA domains comparison, gene structures and conserved motif analysis. We then analyze the gene duplication and synteny between the genomes of wheat and Arabidopsis, rice and barley, which provided insights into evolutionary characteristics. In addition, expression patterns of *TaGATAs* were analyzed, and they showed obvious difference in diverse tissues and abiotic stresses.

**Conclusion:**

In general, these results provide useful information for future *TaGATA* gene function analysis, and it helps to better understand molecular breeding and stress response in wheat.

**Supplementary Information:**

The online version contains supplementary material available at 10.1186/s12870-022-03733-3.

## Introduction

Plants face many environmental challenges during development, and they must optimize growth to adapt to all kinds of environmental condition including abiotic and biotic stress. During the long-term evolution, many kinds of plants have evolved a range of protective mechanisms in response to various environmental stress, among which transcriptional regulations play a dominant role [1]. Transcription factors (TFs) are vital modulators to control gene expression level via specifically binding to promoter region of the downstream gene, thus influencing or regulating a lot of important biological processes, including cellular morphogenesis, signaling transduction, and environmental stress responses [2, 3]. In plants, many well-known transcription factor families have been found, such as GATA (GATA-binding factor) [4], WRKY [5], MYB [6], DREB (Dehydration-responsive element-binding protein) [7], bZIP (Basic region-leucine zipper) [8], and MADS-box [9].

GATAs are a class of DNA binding proteins widely existed in fungi, animals and plants, which belongs to a member of type IV zinc finger, and the DNA binding domain of which is consist of a basic region with C-X_2_-C-X_17–20_-C-X_2−_C form [4]. The GATA proteins could modulate the transcription level of their target genes by recognizing and binding to the (T/A)GATA(A/G) sequences of genes promoter. The first GATA factor NTL1 was found in tobacco (*Nicotiana tabacum*), which was a homolog of NIT2 form *Neurospora crassa* [10]. The GATA family gene was successively identified in a number of plants, such as *Arabidopsis thaliana* [4]*, Oryza sativa* [11, 12], *Glycine max* [13, 14], *Brachypodium distachyon* [15, 16], *Capsicum annuum* [17], *Cucumis sativus* [18], *Gossypium genus* [19] and so on. In plants, all GATA factors are featured with one single zinc finger domain with 18 or 20 residues. In general, the GATA family were classified into four subfamilies in *Arabidopsis thaliana,* as subfamily I, II, III and IV, in terms of phylogenetic analysis, DNA binding domains and gene structures [4].

With the rapid development of Next Generation Sequencing (NGS), GATAs family have been found both in monocots and dicots. There were 30 *GATA* genes in *Arabidopsis*, 28 in rice, 35 in apple, and 64 in soybean according to Genome-wide analyses [4, 13, 20]. Plant GATA TFs have various roles like the chloroplast development [21], photosynthesis and growth [22, 23], seed dormancy [24], host immune response [25], Grain shape [12], and abiotic stress [18, 26, 27]. In Arabidopsis*, GATA12* are involved in primary seed dormancy [24]. SWI2/SNF2 ATPase BRM could associate with GNC (GATA, NITRATE-INDUCIBLE, Carbon metabolism Involved) to regulate SOC1 (Suppressor of Overexpression of Constans 1) expression and control bloom time in Arabidopsis [28]. In rice, Over-expressed *OsGATA12* lead to reduction of leaf and tiller numbers, thus affecting yield-related characters [23]. OsGATA7 regulated architecture and grain shape by mediating brassinosteroids content [12]. In poplar, *PdGATA19* was responsible for photosynthesis and growth [22]. In soybean, low nitrogen treatment led to the obvious repression of *GATA44* and *GATA58* in seedlings [13]. Given the importance of GATA in plants, the above reports manifest that GATA TFs are needed to conduct a comprehensive assessment in development, growth and stress response.

Wheat is the second major cereal crop in the world. Hence, it is important to conduct genetic and physiological research. Many wheat *GATA* genes have been found and functionally characterized. For example, over-expressing TaZIM-A1 postponed flower time and led to the reduction of thousand seed weight [29]. Liu et al. [25] reported that plants of over-expressed *TaGATA1* showed high resistance to *Rhizoctonia cerealis* in wheat. In spite of this, the function of GATA factors defined remains very little in wheat. In the current study, 79 candidate *TaGATA* genes were identified based on the bioinformatic analysis of wheat genome. Generally, we performed a genome-wide analysis in wheat *GATA* genes, such as phylogeny, conserved motifs, gene structures, chromosomal distribution, and expression profiles of *GATA* genes in different tissues and diverse abiotic stresses.

## Materials and methods

### Identification of *TaGATA* genes in wheat

Gene and protein sequences were obtained from the Ensemble Plants database (http://plants.ensembl.org) [30]. To identify the candidate *TaGATA* genes, we used a Hidden Markov Model (HMM) to search the protein database in wheat genome by HMMER3.0 [31], in which the profiles of the GATA protein domain, PF00320, were used as queries with default parameters. Within the same gene ID, we left the longest transcript sequence, and incomplete sequence without start or termination codon were discarded. Then, we used Pfam tool with e-value <e^− 20^ [32] and Conserved Domain Database (CDD) to analyzed the left sequence. Ultimately, 79 *TaGATA* genes were identified. Furthermore, ExPASy tool (http://www.expasy.ch/tools/pi_tool.html) was used to calculate amino acids number, molecular weights (MW) and isoelectric point (pI) [33].

### Phylogenetic analysis of TaGATAs

Sequence alignment of 79 TaGATA protein was conducted using ClustalW [34]. We used MEGA 7.0 to construct Evolutionary tree by the Neighbor-Joining (NJ) method [35], with the following parameters: poisson model, pairwise deletion and 1000 bootstrap replications. The phylogenetic tree was further beautified using the iTOL (https://itol.embl.de/) [36].

### Chromosomal location and gene duplication

*TaGATA* genes localization on chromosome was visualized by MapChart tools (v2.3.2) [37]. Syntenic relationship of the orthologous *GATA* genes between *Triticum aestivum* and *other species*, including *Arabidopsis thaliana, Oryza sativa and Hordeum vulgare*, were analyzed by the MCScanX software [38]. We then used KaKs_Calculator 2.0 to calculate non-synonymous (Ka) and synonymous (Ks) substitution of each duplicated *TaGATA* gene [39]. Formula T = Ks/2R was used to assess Divergence time, where R is 1.5 × 10–8 synonymous substitutions per site per year [39].

### Gene structures and protein motifs analysis

The exon/intron organization of *TaGATA* genes was identified using the Gene Structure Display Server (GSDS) tool (http://gsds.cbi.pku.edu.cn/) [40]. The Multiple Expectation Maximization for Motif Elicitation (MEME) online program (http://meme.sdsc.edu/ meme/itro.html) was performed to identify conserved motifs of TaGATA proteins [41]. The exon-intron structure and conserved motif of TaGATA was examined by TBtools [42] and GFF3 database obtained from Ensemble Plants.

### Cis-elements in the promoter of *TaGATA* genes

Promoter sequences (− 1500 bp) of *TaGATA* gene was obtained from the wheat genome sequence, and cis-element of promoter region was analyzed using PlantCARE software (http://bioinformatics.psb.ugent.be/webtools/plantcare/html/) [43]. The full graphics of Cis-elements were annotated by TBtools [42].

### Gene expression analysis

The specific expression patterns of *GATA* gene from various tissues in the wheat Chinese spring were obtained from Wheatomics (http://202.194.139.32/) [44]. The gene expression values are represented by transcript per Kilobase of exon per million reads mapped (TPM). The average expression level of three biological replicates was calculated and used to show their expression patterns in each tissue. The data were normalized to expression level in roots. Furthermore, transcriptome data under abiotic stress were also obtained from Wheatomics. The genes with log2 ratio ≥ 0.5 and log2 ratio ≤ − 0.5 were regarded as differentially expressed genes (DEGs). A heatmap of expression pattern profile on log2 (TPM + 1) and log2 fold change scale was conducted by TBtools [42, 45].

### Plant growth and treatment

The hydroponic experiment was conducted in a greenhouse at Qingdao Agricultural University, Qingdao, China. Wheat cultivar “Chinese Spring” were used in this study. Chinese Spring is a well-known wheat variety, which has been widely used in wheat genetics research [44]. All the testing wheat seeds were harvested in the summer of 2020 at the Jiaozhou Experimental Station, Qingdao Agricultural University, Jiaozhou, Shandong, China. Seeds of wheat were treated with 3% H_2_O_2_ for 10 min, rinsed seven times with distilled water. The seeds were sown in a controlled environment with a day-night temperature of 22 ± 3 °C on moist filter papers. After germination, uniform seedlings were transferred to 2 L pots containing 1.5 L basic nutrient solution (BNS). On the seventh day after transplanting, PEG and NaCl were added to the containers to form three treatments: BNS (control), BNS plus 10% PEG and BNS plus 100 mM NaCl. The composition of BNS was (mg L^*−* 1^): (NH_4_)_2_SO_4_, 48.2; MgSO_4_, 65.9; K_2_SO_4_, 15.9; KNO_3_, 18.5; Ca(NO_3_)_2_, 59.9; KH_2_PO_4_, 24.8; Fe-citrate, 5; MnCl_2_ 4H_2_O, 0.9; ZnSO_4_ 7H_2_O, 0.11; CuSO_4_·5H_2_O, 0.04; HBO_3_, 2.9; H_2_MoO_4_, 0.01. The solution pH was adjusted to 5.8 *±* 0.1 with NaOH or HCl, as required. Plant leaves and stems were sampled on the seventh day after transplanting. Plant roots samples for RNA isolation were collected 6 h after PEG and NaCl treatment. All samples were stored at *−* 80 °C for downstream analysis.

### Quantitative RT-PCR validation

Quantitative real-time PCR (qRT-PCR) was performed by a QuantStudio3 PCR system (Thermo, USA) [46]. First-strand cDNA synthesis was performed using the PrimeSciptTM RT reagent Kit (Takara, Japan), followed by qRT-PCR using a SYBR Green Supermix (Takara, Japan) with *TaActin* as a reference. The total PCR volume was 10 μl. The reaction process was 94 °C denaturation for 30 s, followed by 40–45 cycles of 94 °C for 5 s, 58 °C for 15 s, and 72 °C for 10 s. Experiments were replicated three times with 2^-ΔΔCq^ relative quantification method. Primers were listed in Table S13.

## Results

### Identification of *TaGATAs* in wheat

In total, 79 GATA family members were identified in wheat. The detailed information of genes and proteins were listed in Tables S1. For example, the amino acid length of 79 TaGATA proteins ranges from 146 to 499. Meanwhile, the molecular weight is ranged from 16.1 to 54.1 kDa. The GATA domain sequences were listed in Table S2.

### Phylogenetic analysis of TaGATA proteins

**Fig. 1:**
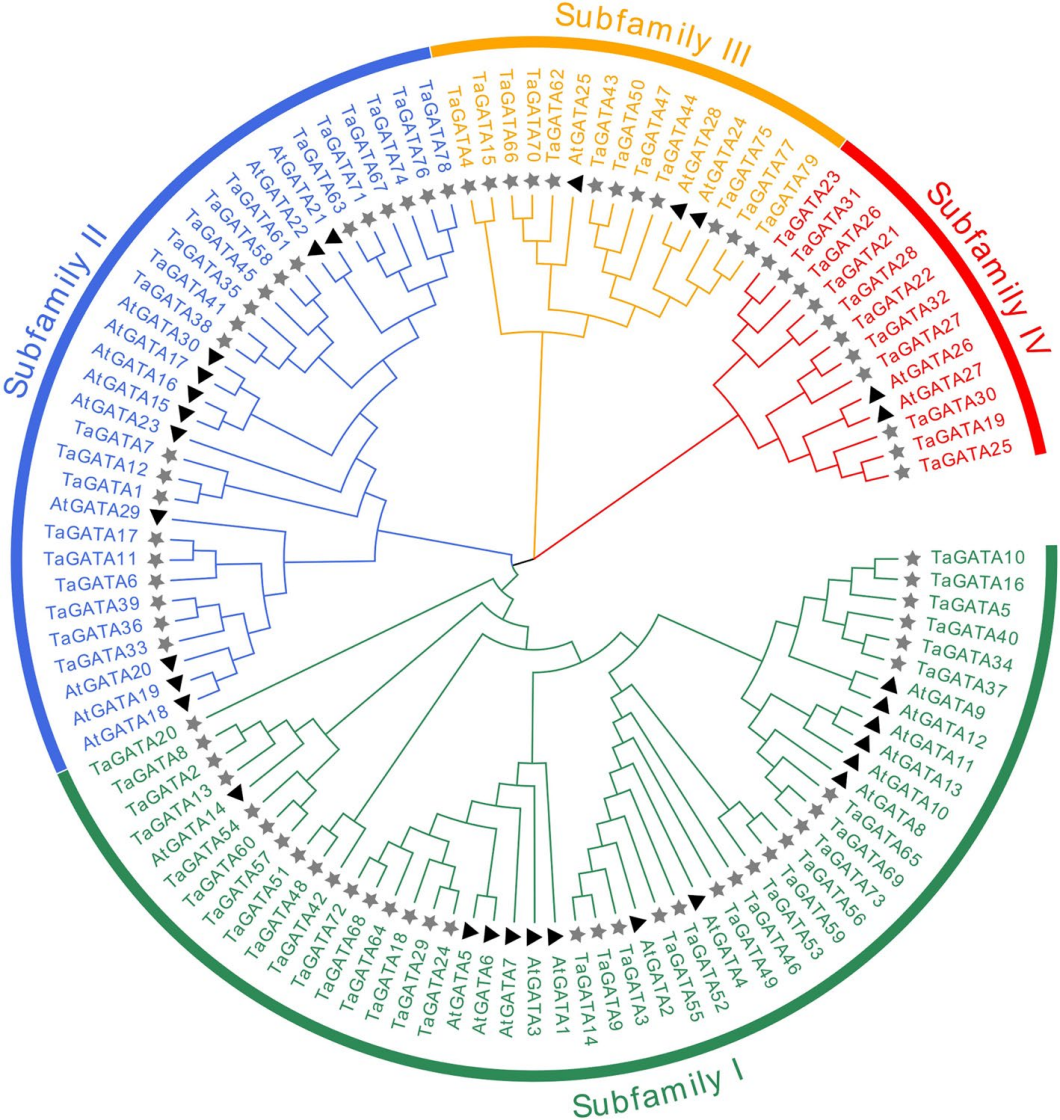
Phylogenetic tree of full-length TaGATA and AtGATA proteins. The different-colored arcs indicate subfamilies of the GATA proteins**.** The tree was constructed using identified 79 TaGATAs (asterisks) in wheat, 30 AtGATAs (triangle) from Arabidopsis. The unrooted Neighbour-Joining phylogenetic tree was constructed using MEGA7 with full-length amino acid sequences and the bootstrap test replicate was set as 1000 times

**Fig. 2:**
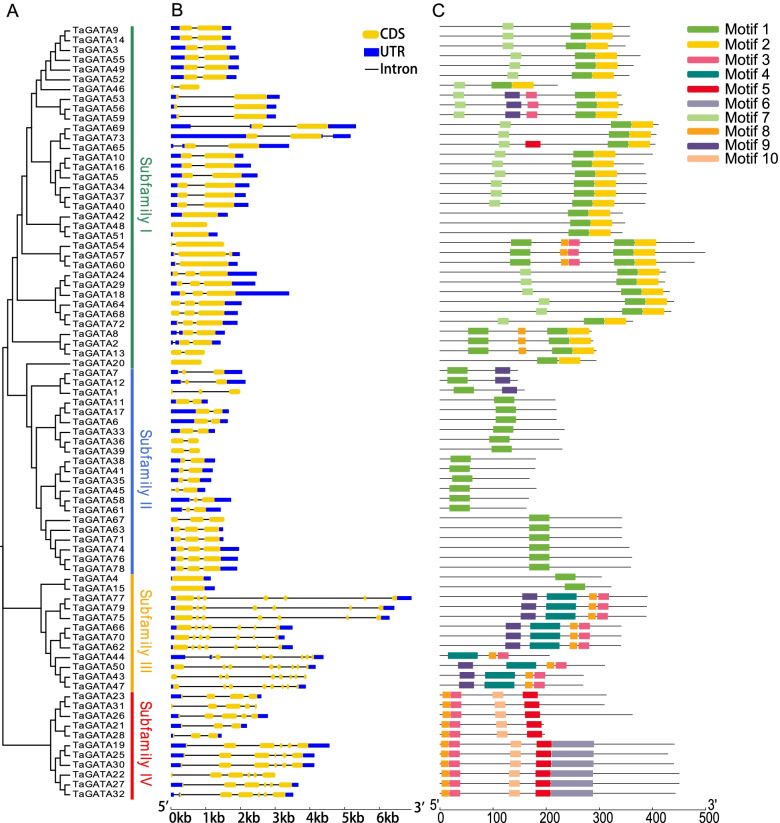
Phylogenetic relationships, architecture of conserved protein motifs and gene structure in *GATA* genes from wheat. **A** The phylogenetic tree was constructed based on the full-length sequences of wheat GATA proteins using MEGA 7 software. **B** Exon-intron structure of wheat *GATA* genes. Blue boxes indicate untranslated 5′- and 3′- regions; yellow boxes indicate exons; black lines indicate introns. **C** The motif composition of wheat GATA proteins. The motifs, numbers 1–10, are displayed in different colored boxes. The sequence information for each motif is provided in Supplementary Files. The length of protein can be estimated using the scale at the bottom

To figure out the phylogenetic relationship of the GATA proteins, we constructed a evolutionary tree in terms of the alignment of 79 wheat TaGATAs and 30 *Arabidopsis* AtGATAs (Fig. 1). The AtGATAs protein sequence were listed in Table S3. It was reported that 30 AtGATA proteins could be categorized into four clusters [4]. On the basis of classification standard used for *Arabidopsis*, the wheat GATA proteins were classified into four group. Group I, II, III, and IV consist of 35, 21, 12, and 11 TaGATA proteins, respectively (Figs. 1 and 2A).

### Gene structure and protein motif analysis of TaGATA

We used the web server GSDS to analyze *TaGATA* genes structures. The results showed that *TaGATA* genes contained one to eight exons unevenly (Fig. 2B). Protein motifs were determined by MEME. In general, 10 conserved motifs were found in TaGATA proteins and considered motifs 1–10 (Figs. 2C). The detailed information of conserved motif were listed in Table S4. In total, 19 of 79 TaGATAs only contain motif 1. Thirty five of 79 TaGATAs contain motif 1 and 2. The motif 1 were primarily presented in subfamily I and II, and the motif 3–10 were detected in the members of group II and IV. In a word, similar gene structures and conserved motifs in the same subfamily forcefully back up phylogenetic analysis for subfamily classifications.

**Fig. 3:**
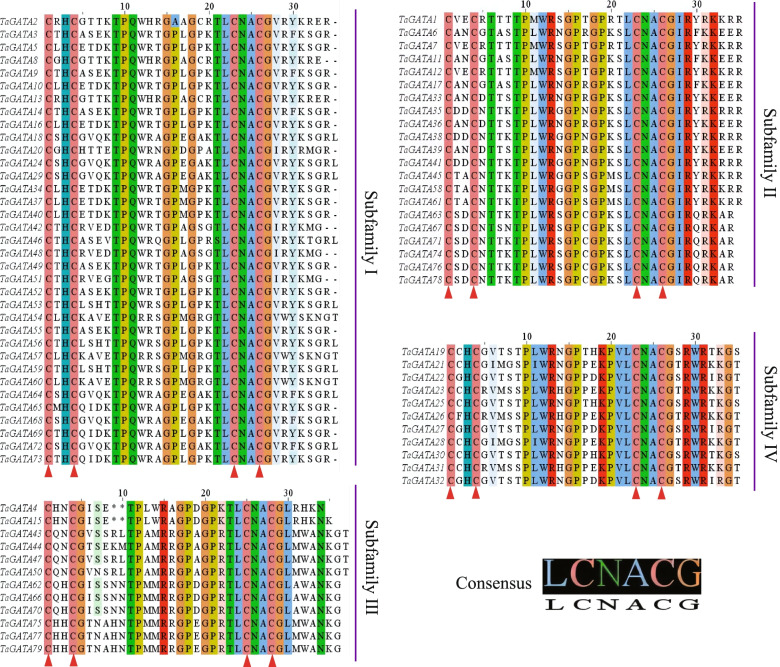
Alignments of GATA domain sequences of the GATA family members in wheat. Highly conserved amino acid positions are marked with letters and triangles at the bottom

In addition, GATA domain analysis showed that TaGATAs in the subfamilies I, II and IV comprised 18 residues in the zinc finger loop between the second and the third Cys residues, while TaGATAs in the subfamily III comprised 20 residues, with the exception of TaGATA4 and TaGATA15 comprised 18 residues. In the GATA domains, many amino acid sites exhibited high conservation, such as LCNACG residues (Fig. 3).

### Chromosomal location and genome Synteny of *TaGATA* genes

**Fig. 4:**
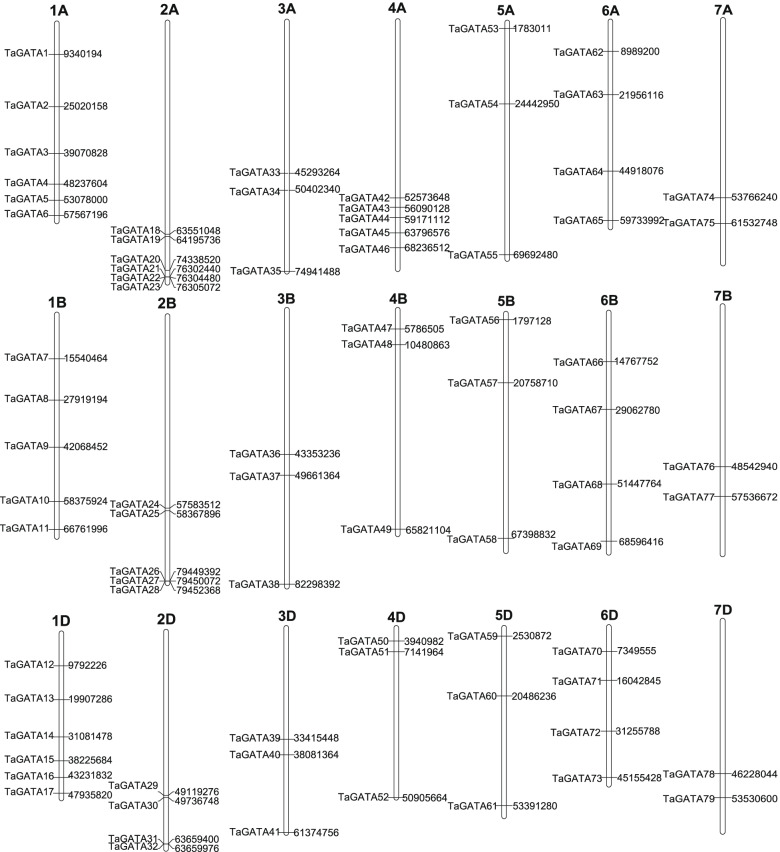
Distribution of *TaGATA* genes in wheat chromosomes. The chromosome numbers are indicated at the top of each chromosome image

The chromosomal distribution of *TaGATA* gene were analyzed. In total, 79 *TaGATAs* were mapped to the wheat genome (Fig. 4). The *TaGATA* genes were evenly located among A (29), B (25), D (25) subgenomes. This was consistent with the finding that a large proportion of *TaGATAs* have three homoeologous sequences distributed on three subgenomes. There were three *TaGATA* genes located on chromosome 3, 5. Six *TaGATAs* could be found on each of chromosomes 1 and 2. Four *TaGATA* genes were located on chromosome 6. Five *TaGATA* genes were distributed on chromosome 4A and three *TaGATA* genes were located on chromosome 4B and 4D. Chromosome 7 carried 2 *TaGATAs* which was the minimum number. With approach of BLAST and MCScanX, we detected 96 segmental duplication events in Ta*GATAs* (Fig. 6; Table S5). All events were almost happened between the different chromosomes. Furthermore, 4 duplication events happened on the AA subgenome, 3 events on the BB subgenome, 4 events on the DD subgenome, and 85 events across AA/BB/DD subgenomes. The above results demonstrate that a number of *TaGATA* genes are likely to appear in the course of gene duplication, and the segmental duplication events could be of great importance in the expansion of *TaGATA* genes in wheat.

The colinearity of *TaGATA* gene pairs between *Hordeum vulgare* genome, *Arabidopsis thaliana* genome and *Oryza sativa* genome was compared. The result exhibited that three and ten *TaGATA* genes exhibited syntenic relationship with *AtGATA* and *OsGATA* genes, respectively (Fig. 7; Table S6 and S7). For example, AT2G45050 showed syntenic relationship with *TaGATA3, TaGATA9* and *TaGATA14* (Table S6). However, 54 *TaGATAs* showed syntenic relationship with *GATAs* in barley (Table S8), implying that these genes may be responsible for the evolution of *TaGATAs* family.

To assess the evolutionary constraints acting, we calculated Ks values, Ka values, Ka/Ks ratios and divergence time of paralogous and orthologous on *GATA* family genes (Tables S9). Ka/Ks ratios were less than 1 in several segmental duplicated *TaGATA* gene pairs, while *TaGATA26/TaGATA31* were more than 1. The results demonstrated that *TaGATA*s family probably have suffered strong purifying selective stress in the course of evolution.

### Cis-elements analysis in *TaGATAs* promoters

**Fig. 5:**
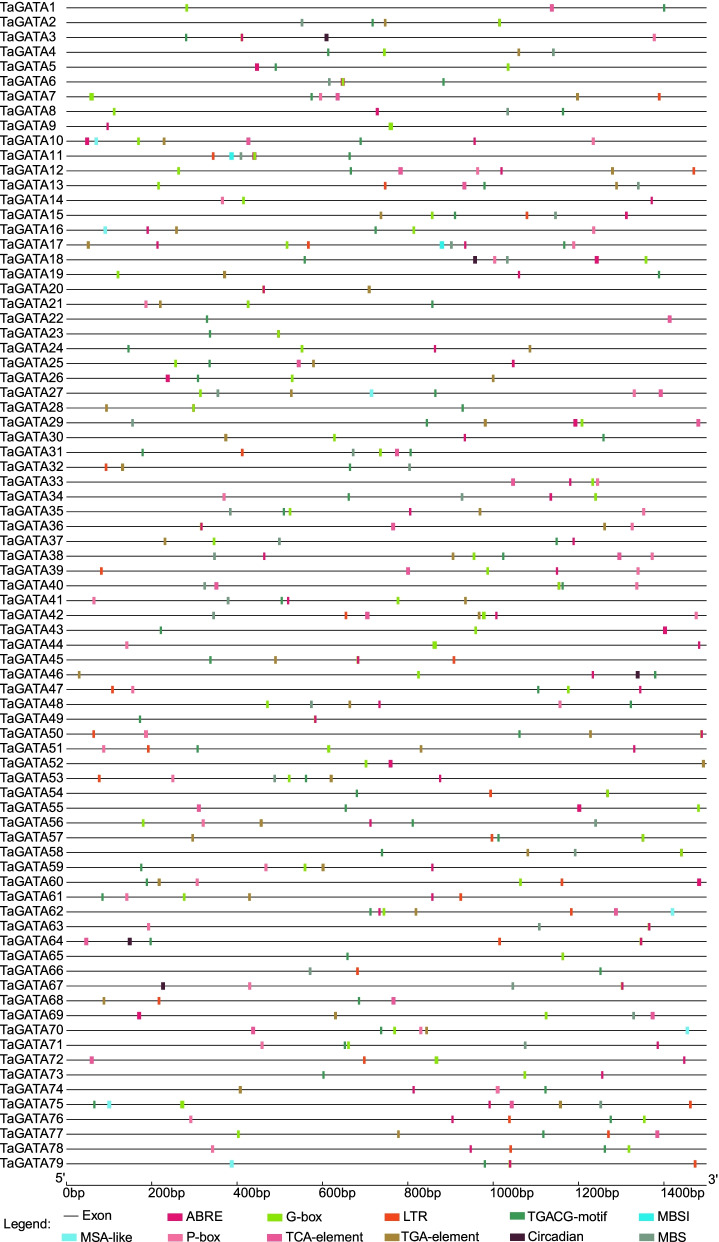
Predicted cis-elements in *TaGATA* promoters. Promoter sequences (− 1500 bp) of 79 *TaGATA* genes were analyzed by PlantCARE. The upstream length to the translation starting site can be inferred according to the scale at the bottom

To explore the underlying function of *TaGATA* genes, we used Plant-CARE to detect the cis-elements in these genes promoter. 79 *TaGATAs* were estimated with cis-elements, such as ABRE, circadian, G-box, LTR, MSA, P-box, TCA, TGA TGACC-motif and MBSI involving in ABA responses, circadian control, light response, low-temperature response, cell cycle regulation, gibberellin response, salicylic acid response, auxin response, MeJA response, drought-inducibility and flavonoid biosynthetic genes regulation (Fig. 5, Table S10). In general, 69 *TaGATA* genes (87.3%) carried ABRE cis-elements, 75 *TaGATA* genes (94.9%) had G-box cis-elements, and 63 *TaGATA* genes (79.7%) carried TGACC cis-elements. In a word, the cis-elements analysis implied that a large portion of *TaGATA* genes are likely to be responded to various environmental stresses.

**Fig. 6:**
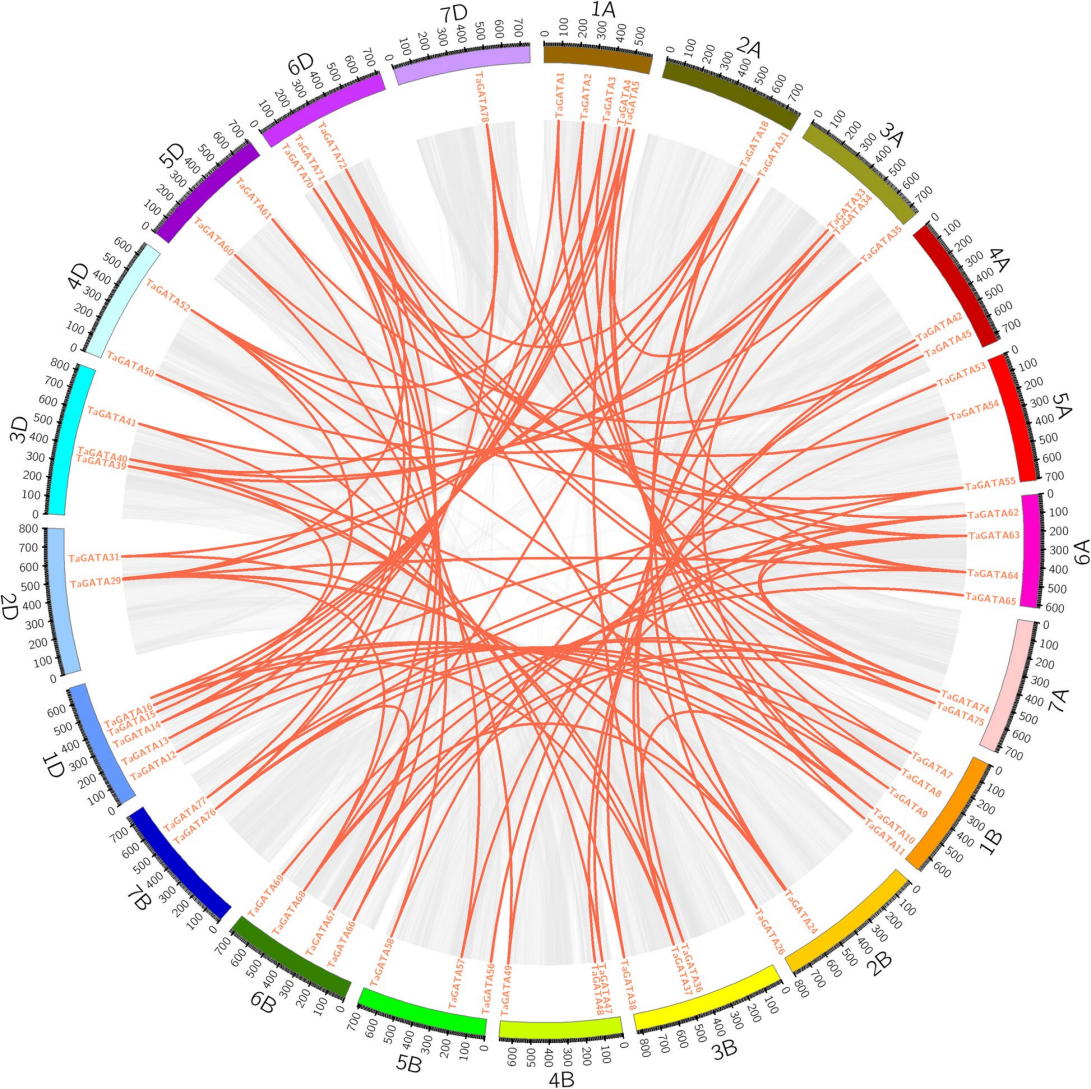
the synteny analysis of *TaGATA* family in wheat. Gray lines indicate all synteny blocks in the wheat genome, and the red lines indicate duplicated *TaGATA* gene pairs. The chromosome number is indicated at the bottom of each chromosome

**Fig. 7:**
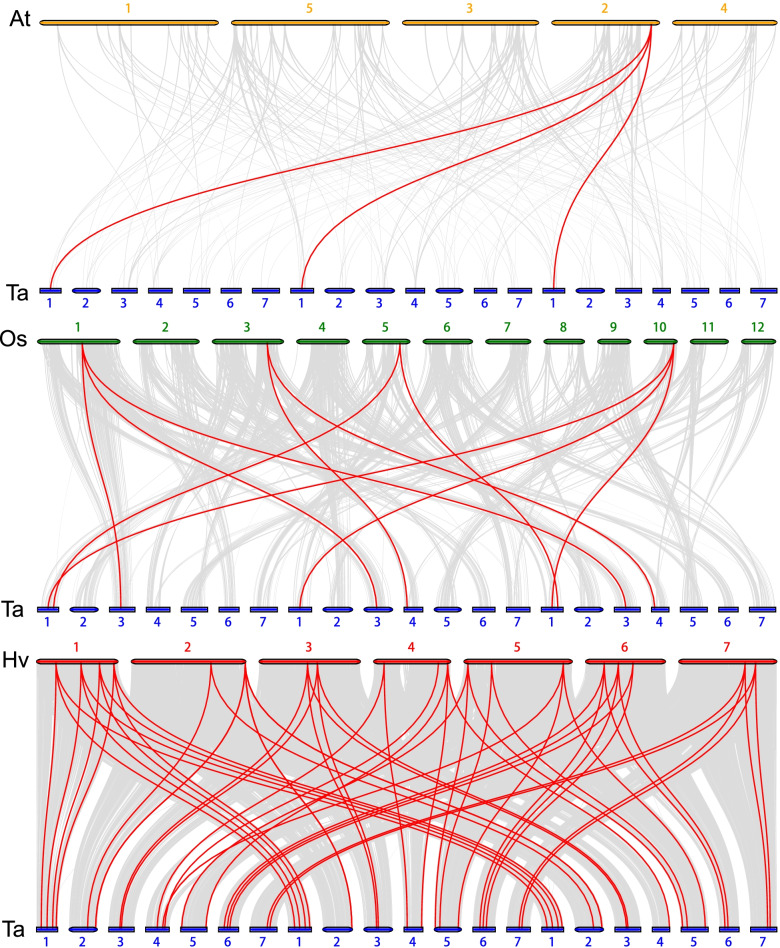
Synteny analysis of *GATA* genes between wheat, Arabidopsis, rice and barley. Gray lines in the background indicate the collinear blocks within wheat and other plant genomes, while the red lines highlight the syntenic gata gene pairs. The specie names with the prefixes, Hv, Ta, At and Os indicate barley, wheat, Arabidopsis and rice, respectively

**Fig. 8:**
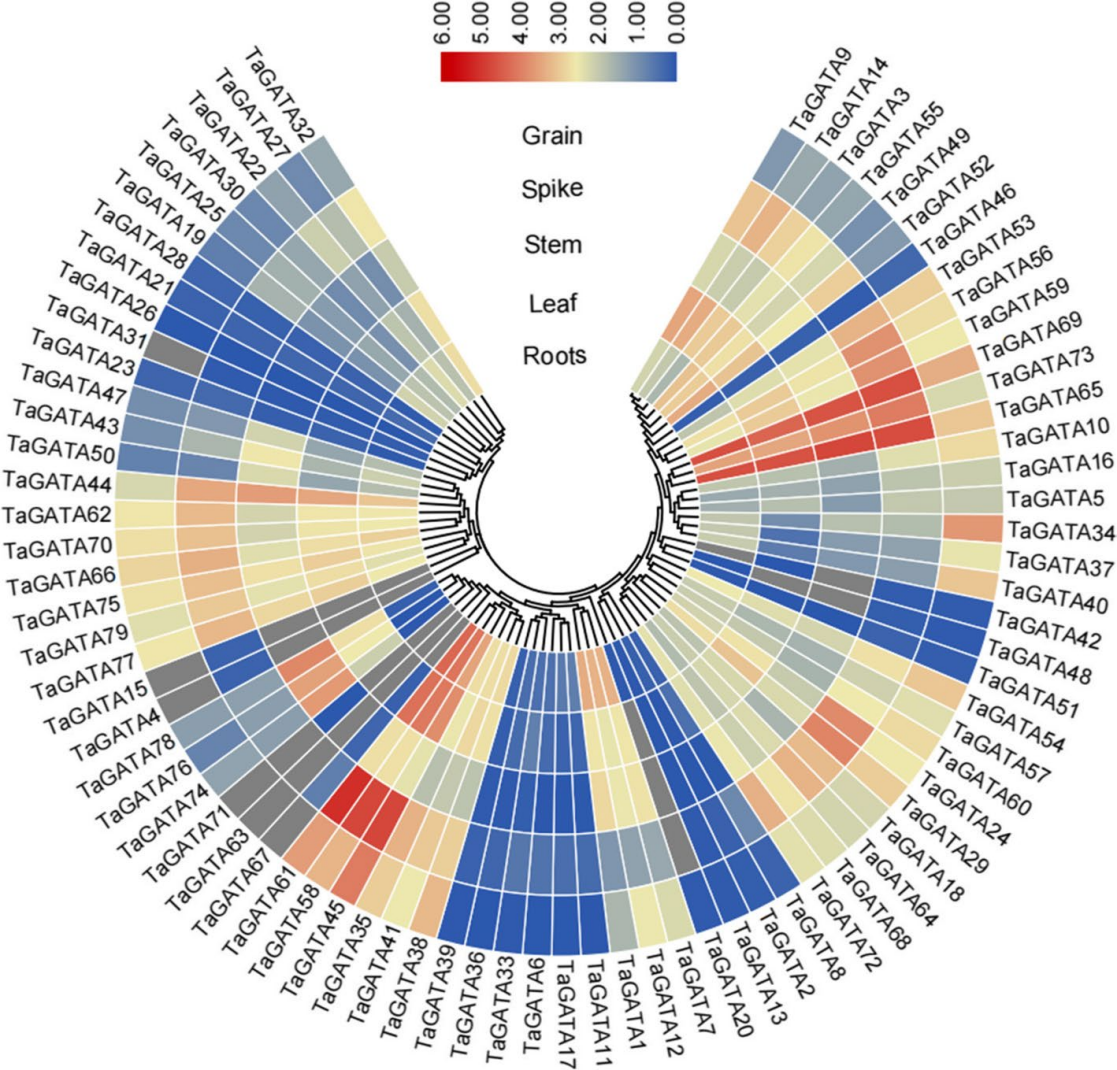
Expression profiles of the *TaGATA* genes in different tissues. Expression data were processed with log2 normalization. The color scale represents relative expression levels from high (red) to low (blue)

**Fig. 9:**
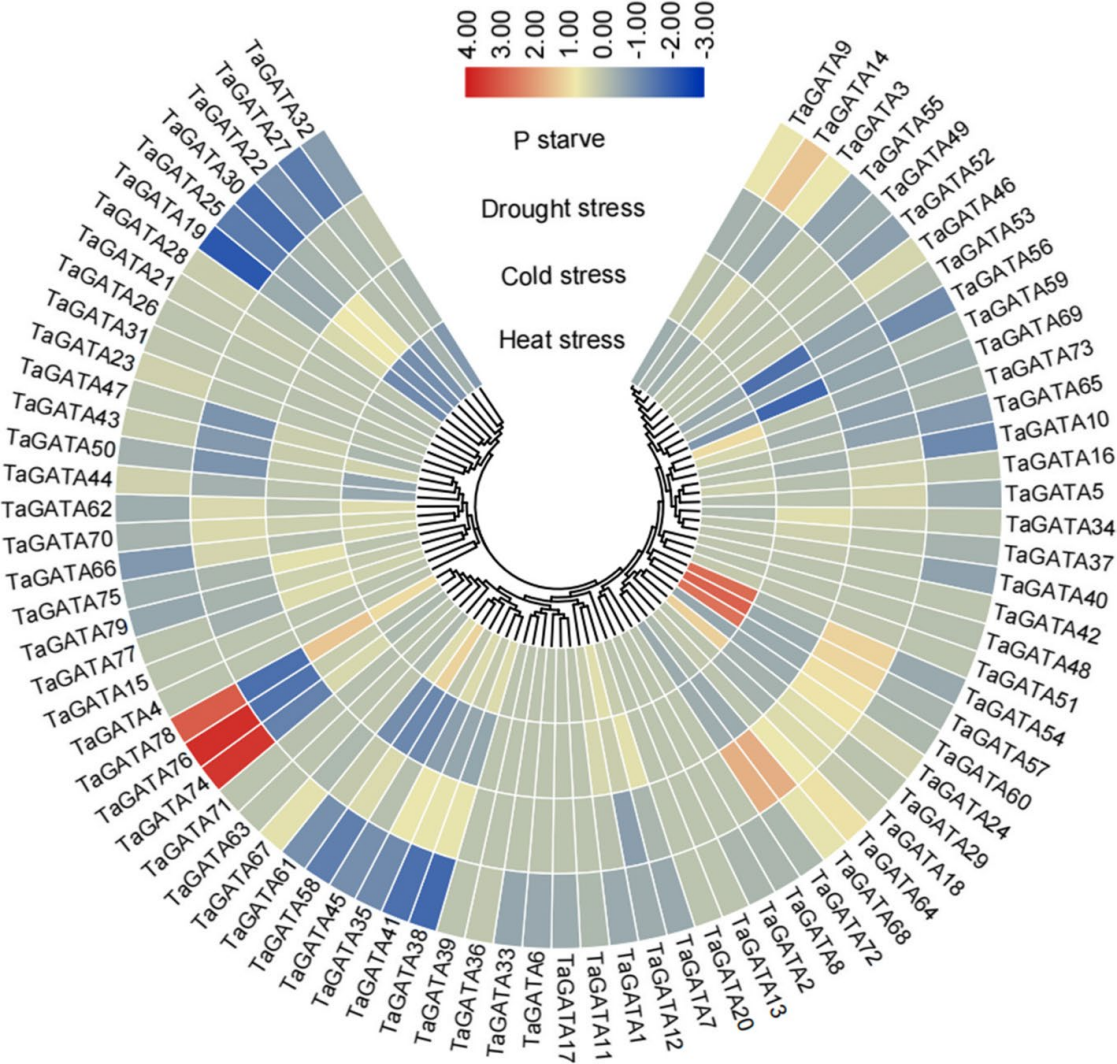
Expression profiles of the *TaGATA* genes under different abiotic stresses. Expression data were the ratio to control values. The color scale represents expression levels from upregulation (red) to downregulation (blue)

### Expression analysis of *TaGATAs* in wheat tissues

The expression patterns of 79 *TaGATAs* in 5 tissues of Chinese spring, including roots, leaves, stems, spikes, and grains, were compared (Fig. 8; Table S11). On the basis of different expression pattern of these genes, they could be classified into two groups. Group 1 include 9 genes, and they were only expressed in some tissues. For example, *TaGATA4* were only expressed in spike, and no expressed in other tissues. Group 2 includes 70 genes, which displayed expression in all tissues analyzed in the current study. Group 2 can be divided into two subgroup. Twelve *TaGATAs* were assigned to the subgroup 1 with high expression levels (log_2_^TPM + 1^ > 2) in all tissues. 10 *TaGATAs* were assigned to the subgroup 2 with low expression levels (log_2_^TPM + 1^ < 0.5) in all tissues. The rest of 48 genes of 70 genes were belong to the subgroup 3. These results implied that *TaGATAs* showed different expression level and genes in the same subfamily also displayed different expression profile.

### Expression patterns of *TaGATAs* under abiotic stress

We analyzed the expression level of *TaGATA* genes under different abiotic stress using the wheat transcriptome data recently published, such as drought, heat, cold stresses and P starvation. Overall, the expression level of *TaGATA* genes significantly changed under diverse abiotic stresses (Fig. 9; Table S12). Several *TaGATA* genes were in response to heat stress or P starvation. For example, the expression level of *TaGATA74, TaGATA76* and *TaGATA78* were extremely increased by P starvation. *TaGATA54, TaGATA57* and *TaGATA60* showed high expression level responding to heat stress. Meanwhile, some *TaGATA* genes were repressed by cold stress, such as *TaGATA53* and *TaGATA59*, or by P starvation, such as *TaGATA19*. In contrast, several *TaGATAs* were not induced by any abiotic stresses. For example, *TaGATA4* and *TaGATA20* displayed almost no expression alteration in response to all analyzed treatments. Instead, several genes displayed opposite expression patterns under different abiotic stress. For instance, *TaGATA78* was extremely induced by all treatments, which showed down-regulation in drought stress, but up-regulation in other treatments. In addition, several *TaGATA* genes were chosen for qRT-PCR to verify the reliability of transcriptome data, and the results were uniform to the sequencing data (Fig. S1, S2).

## Discussion

Transcription factors take a vital regulatory role in plant growth and development. They are the key links in modulating many kinds of physiological activities. Thus far, the GATA family has been reported in a number of plant species, such as Arabidopsis, rice [4], maize [47], apple [20], and *Brassica napus* [48]. The gene structures, expression profile, characteristic features and functions have already been reported in some *GATA* genes. Nevertheless, a genome-wide analysis of the *GATA* family genes have not yet been reported in wheat (*Triticum aestivum* L.). In the present study, 79 members of *TaGATA* genes were found in the *Triticum aestivum* genome, which were identified as *TaGATA1* to *TaGATA79* based on their chromosome location (Fig. 1; Table S1). *TaGATAs* classified into four subfamilies showed obvious difference in genetic structures and expression patterns (Fig. 1 and Fig. 2; Table S11 and Table S12). The current study gives a valuable information for future functional characterization of *GATA* genes and it contributes to increase adaptive capacity when plants subjected to abiotic stress.

In plants, *GATA* genes showed low conservation in their exon/introns structures. In wheat, exons number in *TaGATA* genes ranges from 1 to 8 (Fig. 2), which is very similar to that of *Brassica napus* (1 to 9) [48] and Arabidopsis (2 to 8), and rice (2 to 9) [4]. Except for the zinc finger, the low level of similarity in flanking sequences suggested that the different subfamilies have appeared by modular evolution through shuffling of exons encoding the zinc finger domains [4]. Large divergences in *TaGATA* gene and protein structures could cause functional differences. For instance, the GATA domain featured with 20 residues in the zinc finger in subfamily III, while other three subfamilies showed 18 residues (Fig. 3; Table S4). The CCT and TIFY domains were particularly existed in the subfamily III, which were found to be responsible for flowering, hypocotyl and root development in *Arabidopsis thaliana* [49]*.* For instance, AtGATA23 modulates the auxin response factors ARF7 and ARF19 and influences the lateral root initiation cell differentiation and root branching pattern [50]. The first GATA factor is identified on the basis of the light and circadian clock-related *cis*-elements in its promoters [51]. Moreover, the *Arabidopsis* GATA factors *AtGATA1, AtGATA2*, and *AtGATA4* have been reported to be associated with light regulation of gene expression and photomorphogenesis [52, 53]. Thus, the function of the GATA factors can be predicted according to the identification of *cis*-elements from their promoter. In this study, *TaGATA75* and *TaGATA77* showed high expression level in most tissues of wheat (Fig. 8, Table S11). Meanwhile, the promoter of these genes had TGA-element involved in auxin-responsive. It suggested the importance of these genes in root development. The subfamily I genes were reported to be associated with plant growth and in response to environmental stresses. In *Arabidopsis*, BME3 (ortholog of TaGATA24) could enhance seed germination capacity [54]. In comparison with wild-type plants, seeds in knockout of BME3 plants were more prone to dormancy and more vulnerable to cold stress. In this study, *TaGATA24* were highly expressed in all tissues, and the promoter of *TaGATA24* had ABRE-element and G-box involved in abscisic acid responsiveness and light responsiveness (Fig. 5; Table S10), which are consistent with our results concerning expression pattern under heat and drought stresses (Fig. 8 and Fig. 9; Table S11 and Table S12). Meanwhile, Ravindran et al. [24] found that RGL2-DOF6 complex modulates GATA12 (GATA subfamily I) to promote seed dormancy in *Arabidopsis*. *GATA* genes in subfamily II may be associated with flowering and also in response to abiotic stresses. Expression pattern analysis exhibit that *GATA* genes respond to diverse abiotic stresses, such as high temperature, salinity, cold, and drought treatments in rice, *Brassica juncea*, *Brassica napus*, *Cucumis sativus*, and pepper [17, 18, 48, 55, 56]. In *Arabidopsis*, *GNC* and *GNL* (ortholog of T*aGATA38*) were associated with germination, bloom and cold stress [49]. In *Brassica napus*, the expression level of *BnGATA2.5* (ortholog of *TaGATA38*) was highly depressed under ABA, drought and cold stresses [48]. In this study, *TaGATA38* was expressed across many tissues, and was down-regulated in cold stress and P starve, and up-regulated in heat and drought stress (Fig. 8 and Fig. 9; Table S11 and Table S12). Meanwhile, the promoter of *TaGATA38* had ABRE-element, G-box, MBS involved in abscisic acid responsiveness, light responsiveness and drought-inducibility (Fig. 5; Table S10), thus showing its strong response to environmental stresses. Moreover, over-expressing *BdGATA13* in Arabidopsis led to darker green leaves, more delayed flowering, and more increased drought tolerance [16]. In rice, over-expressing *OsGATA16* and *OsGATA8* enhances cold and drought tolerance, respectively [27, 57]. Over-expression of *SlGATA17* increases drought tolerance in tomato [26]. In this study, *TaGATA54*, *TaGATA57* and *TaGATA60* were increased in heat stress, but decreased in cold stress and P starve. Our qRT-PCR analysis showed that the expression of *TaGATA60* were up-regulated under salt stress, but no response under drought stress (Fig. S1), suggesting that *TaGATA60* could be a functional gene in response to salt stress. However, GATA subfamily IV have known very little so far. Here, Expression pattern analysis showed that *TaGATA19, TaGATA22* and *TaGATA25* were down-regulated in response to heat stress and P starve (Fig. 9; Table S12). Salicylic acid and jasmonic acid have been reported to play important roles in plants responding to abiotic stress [58, 59]. In this study, the promoter of *TaGATA19, TaGATA22* and *TaGATA25* had TGACG-motif and TCA-element involved in MeJA-responsiveness and salicylic acid responsiveness (Fig. 5; Table S10), suggesting subfamily IV of *TaGATA* may be also associated with abiotic stress.

In general, we conducted a comprehensive characterization of GATA family genes in wheat. All these results provide a basis information for manipulating *GATA* genes and facilitate marker-assisted breeding in wheat. Nevertheless, functional identification is necessary for further study to uncover the exact functional characteristic of *TaGATA* genes.

## Supplementary Information

**Additional file 1.**

**Additional file 2.**

## Data Availability

All data analyzed during this study are included in this article and its Additional files.
