# Supplementary material for: Genome-wide identification and characterization of GATA family genes in wheat
Source: BMC Plant Biol. 2022 Jul 27;22:372. doi: 10.1186/s12870-022-03733-3 (PMC9327314; doi:10.1186/s12870-022-03733-3)
Supplement: Supplementary file 2 — Additional file 2. [file 12870_2022_3733_MOESM2_ESM.docx]

Fig. S1. Expression profiles confirmation of twelve genes by qRT-PCR in Chinese Spring under drought stress and salt stress. Roots were sampled after 6 h 10% PEG and 6h 100mM NaCl exposure in the growth cabinet. The relative transcriptional level was analyzed using 2-^ΔΔCq^ method, and the transcriptional level under control was normalized as 1.00. The experiments were performed in triplicates, and values are the mean ± SE.

Fig. S2. Expression profiles confirmation of six genes from various tissues by qRT-PCR in Chinese Spring. Roots, leaf and stem were sampled on the seventh day after transplanting. The relative transcriptional level was analyzed using 2^-ΔΔCq^ method, and the transcriptional level from root was normalized as 1.00. The experiments were performed in triplicates, and values are the mean ± SE.
